# Circulating Tumour Cells as Potential Biomarkers for Oral Squamous Cell Carcinoma

**DOI:** 10.3390/molecules31071145

**Published:** 2026-03-30

**Authors:** Mzubanzi Mabongo, Talent Chipiti, Rodney Hull, Lindokuhle Sibiya, Boitumelo Phakathi, Zodwa Dlamini

**Affiliations:** 1 Department of Otorhinolaryngology, University of KwaZulu-Natal, 719 Umbilo Road, Congella, Durban 4001, South Africa; mzubanzi.mabongo@up.ac.za (M.M.); sibiyal1@ukzn.ac.za (L.S.); 2 SAMRC Precision Oncology Research Unit (PORU), DSI/NRF SARChI Chair in Precision Oncology and Cancer Prevention (POCP), Pan African Cancer Research Institute (PACRI), University of Pretoria, Hatfield, Pretoria 0028, South Africa; talent.chipiti@up.ac.za (T.C.); rodney.hull@up.ac.za (R.H.); 3 Department of Surgery, University of KwaZulu-Natal, 719 Umbilo Road, Congella, Durban 4001, South Africa

**Keywords:** circulating tumour cells, oral squamous cell carcinoma, liquid biopsy, biomarkers, epithelial–mesenchymal transition, multimodal diagnostics, tumour heterogeneity, precision oncology, treatment monitoring

## Abstract

This review evaluates the emerging role of circulating tumour cells (CTCs) as clinically meaningful, minimally invasive biomarkers for oral squamous cell carcinoma (OSCC). Despite advances in management, OSCC continues to demonstrate high morbidity and mortality, largely due to late diagnosis and the absence of validated biomarkers for early detection or real-time monitoring. Conventional diagnostic tools, tissue biopsy, and imaging provide only static snapshots and fail to capture tumour heterogeneity or evolving biological behaviour. CTCs offer a novel and significant opportunity to address these limitations. Key findings from recent studies highlight that CTC enumeration correlates with tumour burden, nodal metastasis, recurrence, and overall prognosis. Molecular and phenotypic characterisation further reveals dynamic traits such as epithelial–mesenchymal transition, stemness, and therapy resistance, providing insights into metastatic potential and treatment failure. Technological advances, including immunocytochemistry, microfluidic capture platforms, PCR-based assays, and next-generation sequencing, have enhanced the sensitivity and specificity of CTC detection and enabled detailed multi-omic profiling. Collectively, evidence suggests that integrating CTC analysis into OSCC clinical workflows could improve early detection, refine risk stratification, personalise therapeutic strategies, and support longitudinal monitoring of disease dynamics. As research progresses, CTC-based diagnostics represent a promising frontier in shifting OSCC management toward more precise, adaptive, and biologically informed care.

## 1. Introduction

The high mortality associated with cancer is largely attributable to late detection and the metastatic behaviour of tumour cells, underscoring the need for early and accurate diagnosis [1,2]. When cancers are identified at an early stage, survival outcomes improve significantly [3]. Advances in molecular oncology have shown that cancer development and progression involve numerous pathways and regulatory mechanisms, many of which can be traced through measurable alterations that serve as biomarkers [4]. As a result, extensive research continues to focus on identifying reliable biomarkers to improve early detection, prognostication, and monitoring.

Oral squamous cell carcinoma (OSCC) remains a prevalent malignancy of the oral cavity and is characterised by local invasiveness with strong potential for regional and distant metastasis via lymphatic and haematogenous dissemination, as demonstrated in recent OSCC biomarker reviews [5]. Clinically, OSCC often manifests as ulcerative or proliferative lesions with necrosis and inflammation affecting sites such as the buccal mucosa, gingiva, tongue, lip, and floor of the mouth. These patterns of clinical presentation are consistent with modern global epidemiological reports showing OSCC as one of the most frequently diagnosed oral malignancies worldwide [6]. Although OSCC may affect any age group, major risk factors include tobacco and alcohol use as well as HPV infection, particularly contributing to increasing OSCC incidence among younger patients [7].

Histopathologically, OSCC comprises malignant proliferations of dysplastic squamous epithelial cells showing invasive growth into surrounding tissues [8]. Diagnosis typically involves clinical examination, imaging modalities, and confirmatory histopathological biopsy. These approaches remain standard across global healthcare systems [9]. Treatment commonly includes surgery, radiotherapy, chemotherapy, or combinations thereof [10]. However, despite therapeutic advancements, OSCC continues to exhibit poor prognosis, particularly when diagnosed late a trend highlighted in both traditional and recent analyses [11].

**Figure 1:**
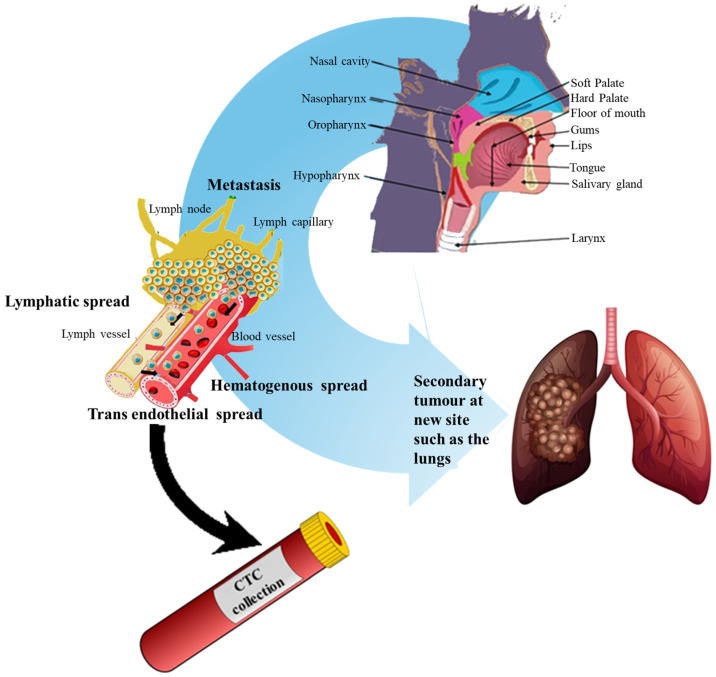
Illustrates the biological trajectory of CTCs: detachment from the primary tumour, intravasation via lymphatic or vascular pathways, circulation, and eventual extravasation at distant sites to initiate metastases. Because these cells can be obtained through minimally invasive sampling, their analysis supports early cancer detection, prognostic classification, and monitoring of treatment response.

Because early detection remains critical, considerable attention has shifted toward minimally invasive diagnostic strategies. Detection of circulating tumour cells (CTCs) has become increasingly important, as CTCs share morphological and molecular features with their tumoural counterparts and allow non-invasive assessment of tumour biology (Figure 1) [12]. The emerging integration of liquid biopsy techniques has transformed cancer monitoring and opened new avenues for precision diagnostics [13]. CTCs enter circulation during early tumour progression and can seed metastases [14]. Elevated CTC counts strongly correlate with advanced cancer stage, metastasis burden, and poorer prognosis in various malignancies [15,16,17].

Dynamic monitoring of CTC levels provides valuable real-time insight into treatment responses. Reductions in CTC levels during therapy often reflect favourable outcomes, whereas persistent or rising counts may indicate resistance or progressive disease, allowing clinicians to adjust treatment strategies accordingly [18]. In OSCC, recent prospective studies confirm that CTC clusters and cell-free DNA levels are associated with tumour burden and metastatic risk [19]. Continuous CTC analysis therefore, has strong potential to improve early detection, prognostic accuracy, and therapeutic monitoring [20].

Traditional diagnostic limitations, including the invasiveness of tissue biopsy and the limited sensitivity of imaging for early micro-metastatic disease, have accelerated the adoption of liquid biopsy approaches [21]. Liquid biopsy enables isolation of tumour-derived analytes such as CTCs (Figure 1), ctDNA, exosomes, and microRNAs from bodily fluids. In OSCC, both blood and saliva serve as valuable sources for biomarker detection [22,23]. Modern systematic reviews emphasise that liquid biopsy biomarkers, particularly tumour DNA and miRNA, show promising diagnostic accuracy and may complement conventional OSCC diagnostic pathways [24]. Figure 1 illustrates the biological trajectory of CTCs, including detachment from the primary tumour, intravasation, circulation, and extravasation at distant sites leading to metastasis.

Recent literature also highlights the relevance of circulating biomarkers in OSCC management. Reviews have underscored that minimally invasive or non-invasive biomarkers, including CTCs, ctDNA, and salivary analytes, represent critical tools for improving early detection, personalising treatment, and overcoming challenges associated with delayed diagnosis [25,26]. Similarly, contemporary OSCC biomarker reviews emphasise the clinical utility and future potential of liquid biopsy approaches for personalised oncology. Collectively, the growing body of evidence demonstrates that the global burden of OSCC necessitates a transition from traditional biopsy-driven diagnostics to biomarker-integrated approaches. Although OSCC currently lacks validated clinical biomarkers, ongoing advances in liquid biopsy, including CTCs, ctDNA, exosomes, microRNAs, and proteomic signatures, offer promising pathways for early diagnosis, molecular stratification, treatment monitoring, and detection of recurrence [27,28].

Building on the overview in Figure 1, liquid biopsy encompasses the analysis of tumour-derived material in body fluids to enable minimally invasive diagnosis and longitudinal monitoring. In OSCC, both blood and saliva are clinically practical matrices: blood supports routine CTC and ctDNA assays, while saliva, by virtue of its proximity to the primary lesion, can be highly informative for nucleic acids, EVs, proteins, and miRNAs, and is increasingly explored alongside blood in OSCC workflows [24,26,29].

Within this framework, CTCs serve several roles. Enumeration is a pragmatic, reproducible measure that correlates with tumour burden and prognosis in solid cancers and is under active investigation in OSCC; importantly, CTC clusters and co-assessment with cfDNA may refine risk stratification [19,30]. Beyond counts, downstream characterisation, spanning morphology and multi-omics (genomics, transcriptomics, proteomics, metabolomics), can illuminate heterogeneity, emergent resistance, and actionable targets, supporting personalised therapy decisions. Routine laboratory methods include immunocytochemistry, PCR-based assays, and NGS panels; these are increasingly coupled with single-cell and spatial approaches to capture intrapatient diversity [6,30].

A complementary and rapidly maturing application is molecular residual disease (MRD) detection using ctDNA, which can anticipate recurrence months before imaging in colorectal cancer and is shaping adjuvant-therapy decisions in prospective cohorts; these advances illustrate the trajectory that head-and-neck oncology, including OSCC, seeks to follow as assay performance and validation improve [31,32]. In breast cancer, ctDNA-guided early endocrine switching for emergent *ESR1* mutations has recently shown a progression-free survival benefit, underscoring how validated liquid-biopsy biomarkers can improve outcomes when tied to adaptive therapy [33]. In prostate cancer, CTC enumeration is prognostic and increasingly used to support treatment stratification in metastatic disease, with expanding liquid-biopsy frameworks [34,35].

By contrast, OSCC currently lacks universally validated blood- or saliva-based biomarkers for routine clinical use, despite strong momentum and encouraging diagnostic accuracy for tumour DNA and miRNA in recent systematic reviews [6,24]. This translational gap explains the field’s pivot from an exclusively empirical model, biopsy plus imaging toward biomarker-integrated care, where CTCs, ctDNA, EVs, and miRNAs are combined to enable earlier detection, risk stratification, therapy monitoring, and recurrence [36,37].

Notably, some biomarkers are already validated in other solid tumours. The CELLSEARCH^®^ CTC test holds FDA clearance for prognostic use in metastatic breast, colorectal and prostate cancers, where CTC counts inform prognosis and serial monitoring; this level of clinical validation is a benchmark OSCC liquid-biopsy programs are working toward [38,39]. As OSCC studies scale up, particularly those integrating CTC enumeration with molecular profiling and saliva–blood multi-analyte panels, the expectation is that assay sensitivity/specificity, standardisation, and multicentre validation will enable routine clinical adoption and, ultimately, better outcomes through earlier intervention and adaptive therapy [6,26,29].

## 2. Biology of Circulating Tumour Cells

### 2.1. Definition, Historical Perspective, and Clinical Recognition

Circulating tumour cells (CTCs) are malignant cells that detach from a primary or metastatic tumour and enter the bloodstream, where they can disseminate to distant organs and seed metastases [15,40]. Historical observations of tumour cells in circulation date back to the 19th century, culminating in Ashworth’s 1869 description of tumour-like cells in blood [41,42]. Clinically, CTCs are validated prognostic biomarkers across several solid malignancies, with large prospective studies demonstrating associations between CTC burden, progression, and survival, and with the CellSearch^®^ platform receiving FDA clearance for enumeration [43,44,45,46].

Beyond acting as “seeds” for metastasis, CTCs can reflect tumour genomic and phenotypic heterogeneity and emerging resistant clones, making them a minimally invasive substrate for molecular profiling [16,47]. Mechanistically, detachment from the primary tumour is often initiated by local degradation of the extracellular matrix (ECM) via matrix metalloproteinases (MMPs), loosening of cell–cell adhesions mediated by downregulation of E-cadherin, and remodelling of the actin-cytoskeleton. These changes are frequently orchestrated by EMT-associated transcription factors such as Snail, Slug, and Twist, which collectively facilitate motility and intravasation into nearby vasculature [47].

**Figure 2:**
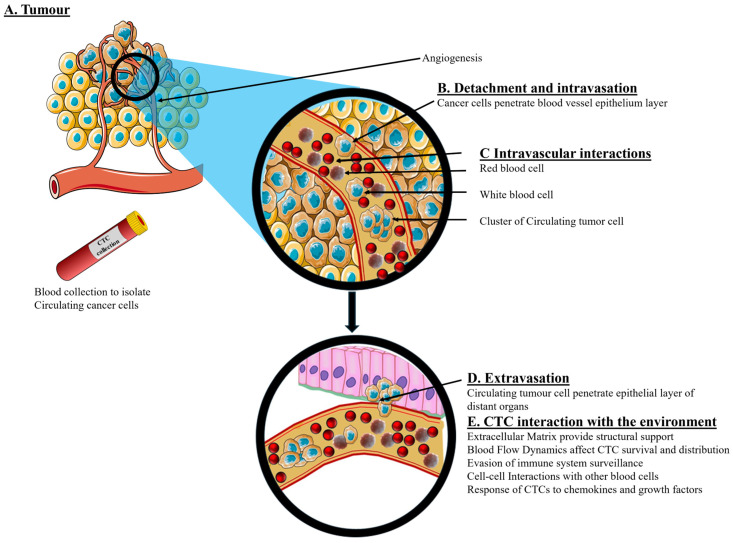
Overview of circulating tumour cell (CTC) biology, intravascular dynamics, and interactions during metastatic progression. (A) Tumour: Primary tumour showing angiogenesis and the release of tumour cells into the nearby vasculature. Blood collection enables the isolation of circulating cancer cells for downstream analysis. (B) Detachment and intravasation: Tumour cells undergo epithelial–mesenchymal transition (EMT), penetrate the endothelial barrier, and enter the bloodstream. (C) Intravascular interactions: CTCs travel within the circulation while interacting with red and white blood cells. Both single CTCs and multicellular CTC clusters may form, influencing survival and metastatic potential. (D) Extravasation: CTCs arrest in distant capillary beds and traverse the endothelial layer to invade secondary organs, initiating metastatic colonisation. (E) CTC interaction with the microenvironment: Upon entering the circulation and during dissemination, CTCs are influenced by extracellular matrix components, blood-flow dynamics, immune surveillance, chemokine gradients, growth factors, and interactions with neighbouring blood cells, factors that collectively modulate CTC survival, distribution, and metastatic competency.

An overview of the biological processes associated with CTC generation, circulation, isolation, and clinical utility is illustrated in Figure 2.

According to many studies, the concentration of CTCs in a patient’s blood sample can be a valuable biomarker for assessing cancer progression. An increase in the number of CTCs can be directly linked to an increase in the patient’s cancer stage [15]. Higher levels of CTCs are found in more aggressive disease states, which is correlated with a higher cancer recurrence rate among patients [20,21]. The concentration of CTCs in the blood is an important clinical prognostic indicator. In some cases, a greater number of CTCs have been directly associated with poorer survival rates in cancer patients, as observed in various solid cancers, such as breast, lung, prostate, and colorectal cancers [48]. Additionally, based on clinical observations, the total number of CTCs in the blood samples of cancer patients decreased significantly following effective treatment. This underscores the potential of monitoring changes in CTC numbers as indicators for physicians to evaluate the response of cancer patients to therapy [49]. According to the aforementioned studies, the counting and monitoring of CTC numbers in specific blood samples are widely recognised as feasible clinical applications. The CellSearch system, whose CTC counting mechanism has received FDA approval for evaluating cancer patients’ prognoses, is particularly noteworthy [16].

In addition to CTC counts, downstream analysis of CTCs provides additional information on cell morphology and multinomics (i.e., genomics, transcriptomics, proteomics, and metabolomics). The analysis of CTC characteristics plays a pivotal role in understanding cancer biology, behaviour, metastasis, and drug resistance [50,51]. CTCs exhibit remarkable cell heterogeneity in terms of genotype, phenotype, and morphology due to the heterogeneity of the primary tumour [52]. The dynamic interplay between CTCs and their microenvironments is crucial and reflects cancer progression, metastasis, and adaptation to environmental changes. The detection of CTC heterogeneity also reflects cancer progression, metastasis, and adaptation to environmental changes [53]. Epithelial–mesenchymal transition (EMT) and mesenchymal–epithelial transition (MET) serves as critical mechanisms in cancer metastasis, affecting CTC phenotypic changes [54]. Even though a deep understanding of the cell characteristics and heterogeneity of CTCs is essential for developing new cancer diagnosis and treatment approaches, these detection techniques are costly, time-consuming, and complicated to implement. Counting CTC numbers and monitoring their dynamic changes have become the current mainstream options for early cancer detection, prognosis evaluation, and therapeutic response monitoring because of their relatively low cost and simple detection process [21]. CTCs are shed from primary tumours into the bloodstream, where they can travel to distant sites and potentially form secondary tumours. Understanding CTC release and circulation mechanisms is crucial for developing effective diagnostic and therapeutic strategies.

### 2.2. Origin and Mechanisms of Dissemination: Invasion, EMT, and Intravasation

CTCs are shed from primary tumours into the bloodstream, where they can travel to distant sites and potentially form secondary tumours. Understanding CTC release and circulation mechanisms is crucial for developing effective diagnostic and therapeutic strategies. Under normal circumstances, the extracellular matrix is a well-organised structure responsible for cellular support, communication between cells, and the environment. The ECM provides anchorage for cells, chemical, and mechanical signals that mediate several cell functions that influence cellular behaviour and morphology [54,55].

During the oncogenic process, this homeostasis between ECM and epithelial cells is disrupted by various factors, such as cancer-associated microRNA (miRNA), which regulate early oncogenic processes, and stiffness of the ECM, which promotes mechano-transduction signals that seem to support the cancer cells, thus promoting survival, proliferation, and invasion. Cancer-associated microRNAs (miRNAs) regulate key oncogenic processes such as ECM remodelling, epithelial-to-mesenchymal transition (EMT), and tumourigenic vascular development, further driving tumour growth [51,52,55]. CTC generation begins as tumour cells acquire cellular plasticity within an evolving microenvironment. The epithelial–mesenchymal transition (EMT) enables motility and invasion through loss of epithelial polarity, junctional disruption, and extracellular matrix remodelling via MMPs [55,56]. EMT is driven by *TGF*-β, *Wnt*/β-catenin, Notch, *ERK/MAPK*, and hypoxia-responsive signalling, with transcription factors such as Snail, Slug, Twist, and *ZEB1/2* repressing E-cadherin and inducing mesenchymal markers [57].

These changes result in the formation of mesenchymal cells with stemness characteristics, the ability to evade immunity, and increased aldehyde dehydrogenases (ALDH) [52,53,54]). Following EMT, tumour cells actively intravasate through the endothelial barrier by forming protease-rich invadopodia that locally degrade basement membranes. This process is facilitated by paracrine signals from stromal fibroblasts and endothelial cells, including *VEGF* and *CXCL12*, which increase vascular permeability. Tumour–endothelial interactions, mediated by integrins (e.g., *αvβ3*, *α5β1*) and selectins, further guide tumour cells into circulation, while the actomyosin contractility machinery enables squeezing through narrow capillaries. Hypoxic regions within tumours amplify these processes by stabilising HIF-1α, which promotes EMT and angiogenesis concurrently [55,56,57,58,59,60,61].

Hypoxia and *TGF-β* can concurrently promote EMT and stem-like traits linked to therapy resistance [58,59]. Following stromal invasion, tumour cells intravasate via protease-rich invadopodia and coordinated tumour–endothelial interactions [60,61].

### 2.3. Survival in the Bloodstream: Shear Stress, Immune Escape, and Clustering

Once in circulation, CTCs face shear stress, anoikis, and immune surveillance. Survival is enhanced by platelet cloaking, which not only protects against NK-cell cytotoxicity but also provides paracrine TGF-β signalling to maintain mesenchymal traits. Additionally, CTCs upregulate anti-apoptotic proteins (*Bcl-2*, *Mcl-1*) and express surface molecules such as *PD-L1* to evade immune detection. CTC clusters benefit from intercellular junctions and cytoplasmic sharing of survival signals, significantly enhancing metastatic competency [61,62,63,64,65,66,67]. Platelet cloaking protects CTCs from NK-cell cytotoxicity and shear forces and promotes adhesion to endothelium; platelet-derived *TGF-β* also reinforces EMT programs [61,62]. Interactions with neutrophils and myeloid cells contribute to immune evasion and pro-metastatic niches [63,64]. 

Although CTC clusters constitute only ~2–5% of CTC events, they exhibit dramatically higher metastatic potential due to oligoclonal cooperation and enhanced survival [49,63,64,65]. These features also increase the likelihood of capillary arrest and extravasation [66,67]. These intravasated cancer cells (CTC) and CTC clusters contain stromal cells and immune components from the original microenvironment that contribute to the heterogeneity of the cluster and enhance its survival [64]. Neutrophils participate in cluster formation around the CTCs, thus suppressing leukocyte activation, and increasing the chances of CTC survival [63,64].

### 2.4. Extravasation and Colonization: MET and Metastatic Outgrowth

At metastatic sites, CTCs adhere to endothelium and penetrate the vessel wall. Partial or full mesenchymal–epithelial transition (MET) facilitates proliferation and colonization [67,68]. At metastatic sites, CTCs arrest via interactions with selectins, integrins, and chemokine receptors (e.g., CXCR4), which guide site-specific extravasation. Extravasation involves cytoskeletal remodelling and transient disruption of endothelial junctions, while MET restores epithelial characteristics that enable colonization. Local stromal cells, ECM composition, and angiogenic factors collectively determine the success of metastatic outgrowth [67,68,69]. Immune evasion, adaptation to foreign microenvironments, and angiogenesis are critical for secondary tumour formation. In many cancers, dormancy precedes macrometastatic outgrowth [69].

### 2.5. Phenotypic Heterogeneity and Major CTC Phenotypes

CTCs exhibit extensive phenotypic heterogeneity that mirrors the diversity of the primary tumour and reflects dynamic transitions driven by epithelial–mesenchymal transition (EMT), microenvironmental pressures, and therapeutic selection. This heterogeneity underpins the complexity of metastasis, influences the efficiency of CTC detection, and affects the clinical interpretation of CTC-based biomarkers. Broadly, CTCs can be described across four principal phenotypic categories, each contributing uniquely to tumour dissemination and clinical outcomes.

(I) Epithelial CTCs represent the classical phenotype and are defined by the presence of epithelial markers including *CK8/18/19*, *EpCAM*, and nuclear DAPI positivity, alongside the absence of the leukocyte marker CD45 [16,70,71]. These cells typically retain strong cell–cell adhesion characteristics and often predominate in early-stage disease. Because most clinically validated enrichment platforms, including the FDA-cleared CellSearch^®^ system, rely on EpCAM-based capture, epithelial CTCs remain the most consistently detected population in clinical practice [46,72].

(II) Mesenchymal CTCs emerge largely as a result of EMT, during which epithelial markers (EpCAM, CKs) are downregulated, and mesenchymal markers such as vimentin and N-cadherin are upregulated [56,73]. EMT equips these cells with enhanced migratory capacity, invasiveness, resistance to shear-stress-induced apoptosis, and improved adaptability within the bloodstream. Mesenchymal CTCs are strongly associated with aggressive disease behaviour, metastatic competency, and poorer prognosis in HNSCC, including OSCC [74]. However, their reduced epithelial marker expression makes them more challenging to detect using conventional EpCAM-dependent methods.

(III) Hybrid or partial-EMT CTCs constitute a particularly important intermediate state characterised by simultaneous expression of epithelial and mesenchymal features. These cells arise from incomplete EMT and retain partial epithelial adhesion while acquiring mesenchymal motility, resulting in exceptional phenotypic plasticity [75,76]. Hybrid CTCs demonstrate resistance to anoikis, high adaptability within circulation, and a strong capacity to revert to an epithelial phenotype (MET) during colonisation at distant sites. Increasing evidence suggests that hybrid CTCs possess higher metastatic potential than fully epithelial or fully mesenchymal CTCs and are frequently enriched in CTC clusters.

(IV) Stem-like CTCs represent a subgroup with tumour-initiating characteristics, expressing markers such as *CD133*, *ALDH1*, *SOX2*, *OCT4*, and *NANOG* [53,77]. These stem-associated phenotypes contribute to recurrence, therapeutic resistance, and long-term metastatic persistence. EMT-related transcription factors, including *Snail*, *Slug*, *ZEB1*, and *Twist*, not only regulate EMT transitions but also activate stemness gene programs, reinforcing CSC-like properties [76]. The presence of stem-like CTCs is increasingly recognised as a key prognostic indicator in several solid tumours.

The diversity of these phenotypes highlights the central role of EMT in shaping CTC heterogeneity. The diversity of these phenotypes highlights the central role of EMT in shaping CTC heterogeneity. Mechanistically, dynamic EMT–MET plasticity allows cells to toggle between epithelial adhesion and mesenchymal motility states, enhancing survival under hemodynamic stress, evasion from immune surveillance, and successful colonization at distant sites. Differential expression of transcription factors, adhesion molecules, cytoskeletal regulators, and stemness genes underpins the functional heterogeneity observed in circulating and metastatic tumour populations [78,79,80]. EMT drives loss of epithelial adhesion, increased motility, resistance to apoptosis, and extracellular matrix remodelling, thereby enhancing the potential [78]. Importantly, CTCs broadly reflect the mutational and transcriptomic landscape of the primary tumour and may capture additional evolutionary changes arising from therapy-induced selection pressures [79,80]. Thus, beyond enumeration, molecular profiling of CTC subtypes provides critical insights into tumour invasiveness, drug susceptibility, and emergent resistance patterns [27].

Nonetheless, significant detection challenges remain. EpCAM-based platforms may under-represent mesenchymal and hybrid CTCs, potentially biasing clinical interpretations [46,81]. This limitation underscores the need for multimodal and marker-independent enrichment strategies capable of capturing the full spectrum of CTC phenotypes.

### 2.6. Abundance, Clustering, and Analytic Implications

Despite their biological and clinical significance, CTCs are exceedingly rare in circulation, typically appearing at only 5–50 cells per 7.5 mL of blood, which contains billions of erythrocytes, leukocytes, and platelets [79,80]. This extreme rarity makes analytical isolation technically challenging and places high demands on enrichment sensitivity. While most CTCs circulate as single tumour cells, a small proportion approximately 2–5% form multicellular aggregates known as CTC clusters, circulating tumour micro-emboli (CTM), or circulating micrometastases [42,73,82]. These clusters typically comprise 3 to 100 tumour cells held together by strong intercellular junctions and often include stromal or immune cells. Although numerically rare, CTC clusters possess remarkably high metastatic efficiency, estimated to be 23–50 times that of single CTCs, owing to collective survival advantages, resistance to shear stress and apoptosis, enhanced extravasation, and preservation of stem-like subpopulations [49,65,83].

The coexistence of single CTCs, hybrid phenotypes, and highly potent CTC clusters underscores the need for sensitive, multimodal enrichment and integrative analytic strategies that combine enumeration with morphological and molecular profiling [16,47]. Advances in high-resolution genomic and transcriptomic technologies have further enhanced the diagnostic value of CTCs, transforming them from mere biomarkers of disease burden into dynamic indicators of tumour evolution, therapeutic response, and emerging resistance.

### 2.7. Relevance to OSCC

In oral squamous cell carcinoma (OSCC), CTC biology mirrors aggressive tumour behaviour: EMT plasticity, immune interplay, and genomic variability correlate with local invasion, nodal spread, and distant metastasis [58,59].

Multiple OSCC and HNSCC studies report associations between CTC positivity or burden and advanced stage, nodal involvement, recurrence, and survival, although variability exists across isolation and detection platforms [19,84,85,86,87,88,89]. Because CTCs closely approximate the dynamic molecular evolution of OSCC, including the emergence of therapeutic resistance, liquid biopsy offers a minimally invasive strategy for risk stratification, disease monitoring, and treatment response assessment [47,90]. 

## 3. Current Detection Methods Used for Circulating Tumour Cells

### 3.1. Overview of Current Techniques for CTC Isolation and Detection

The isolation and enrichment of CTCs present various challenges, with the existence of various phenotypes being one of the challenges. These include individual cells, clusters, the expression of epithelial markers, and epithelial, mesenchymal, and cancer stem cells [91]. This heterogeneous nature of CTCs poses challenges in their isolation and enrichment techniques. Rushton et al. (2020) described stringent criteria for CTC detection and enumeration technologies as follows: “high detection and recovery rates; accurate throughput and enumeration; ability to detect heterogeneous types of CTCs; minimal preprocessing of liquid biopsy; and fully automated and wide clinical applicability in various malignancies [92,93].

**Figure 3:**
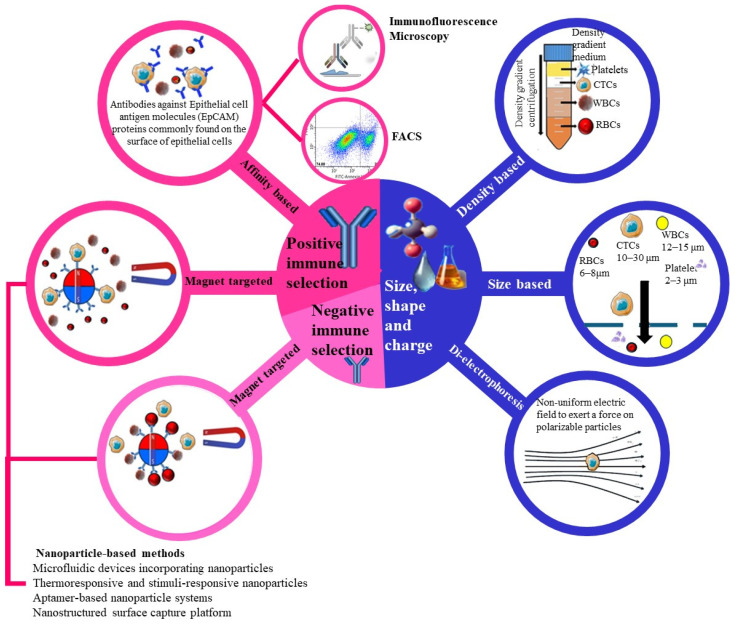
Technology for the detection and enrichment of CTCs. This figure illustrates immunological and biophysical methods for detecting and isolating circulating tumour cells (CTCs). Immunological approaches include positive and negative selection using antibodies or magnetic beads targeting epithelial markers. Biophysical techniques exploit differences in size, density, and electrical properties, such as density gradient centrifugation, size-based filtration, and dielectrophoresis. These methods enhance CTC detection for cancer diagnosis and monitoring.

CTCs are cancer cells that detach from primary tumours and then circulate in the bloodstream as potential seeds for metastasis. Detecting and analysing CTCs offers invaluable insights for early cancer detection, prognosis, and treatment monitoring. Various advanced techniques have been developed for the isolation and detection of CTCs, each with its own set of advantages and limitations. Since CTCs exist in various forms, such as epithelial, mesenchymal, mixed epithelial–mesenchymal, cluster (microembryonic) and stemness forms, the success of detection depends on the form of CTC that is targeted. For example, the CellSearch technique captures epithelial CTCs, as they express the epithelial marker EPCAM [46]. However, negative immunomagnetic techniques are more likely to capture all forms of CTCs because they target normal blood cells. The following is a detailed overview of current methods for CTC isolation and detection (Figure 3). The detection and isolation of CTCs is achieved via the biophysical properties and immuno-affinity of these cells [93].

Immunological approaches involve both positive and negative immune selection. In positive selection, antibodies targeting epithelial markers such as EpCAM specifically bind to CTCs, facilitating their capture. Negative selection, on the other hand, utilises magnetic beads to deplete non-CTC blood cells, indirectly enriching the CTC population.

Biophysical isolation methods rely on the distinct physical properties of CTCs, including size, density, and electrical properties. Density gradient centrifugation, as shown in Figure 3, separates CTCs based on their unique buoyant density compared to other blood components. Size-based filtration exploits the relatively larger size of CTCs (10–30 µm) compared to red blood cells (6–8 µm) and white blood cells (12–15 µm). Dielectrophoresis, also depicted in Figure 3, applies a non-uniform electric field to separate CTCs based on their polarizability. These technologies, as outlined in Figure 3, play a crucial role in improving the detection, isolation, and study of CTCs, aiding in cancer diagnosis, prognosis, and treatment monitoring.

#### 3.1.1. Density Gradient Centrifugation

Density gradient centrifugation is a widely recognised technique for the separation of cells based on their density differences, particularly in the context of isolating circulating tumour cells (CTCs) from blood samples. This method involves layering a blood sample over a density gradient medium, such as Ficoll-Paque, and then centrifuging the sample. The centrifugal force causes the various cellular components’ red blood cells, white blood cells, and CTCs, to stratify into distinct layers according to their respective densities, thereby facilitating the isolation of CTCs from other blood components [94]. The principle behind density gradient centrifugation relies on the unique density of CTCs compared to other blood cells. When subjected to centrifugal force, these cells migrate through the gradient until they reach an equilibrium point, forming discernible bands. This method is favoured in many laboratories due to its simplicity and cost-effectiveness, as it does not require sophisticated equipment beyond standard laboratory centrifuges [94,95]. For effective CTC isolation, blood samples are typically mixed with anticoagulants to prevent clotting, layered over the gradient medium, and centrifuged at speeds ranging from 400 to 600× *g* for 30 to 40 min. Post-centrifugation, CTCs can be extracted from the specific interface using a pipette for further analysis [94,96]. Despite its advantages, density gradient centrifugation has notable limitations. One significant drawback is its inability to effectively differentiate between CTCs and other blood cells that share similar densities, which can lead to contamination in the isolated CTC fraction. This contamination can compromise the purity of the CTCs, which is critical for accurate downstream analyses [94]. Additionally, there is a risk of CTC loss during the layering and collection processes, which can adversely affect the yield and accuracy of subsequent analyses. The manual nature of these steps can also introduce variability and require a careful technique to minimise errors [95]. To enhance the specificity and efficiency of CTC isolation, researchers have explored advancements that combine density gradient centrifugation with other techniques. For instance, integrating immunomagnetic separation or microfluidic technologies can improve the purity and yield of isolated CTCs. Following initial enrichment through density gradient centrifugation, additional isolation steps can selectively target CTCs based on surface markers or other distinguishing properties [97]. Moreover, the automation of the fraction collection process can reduce variability and improve the reproducibility of results, while the development of enhanced gradient media aims to achieve better separation of different cell types. While density gradient centrifugation remains a foundational technique for isolating CTCs, its limitations necessitate the incorporation of supplementary methods and technologies to enhance specificity and purity. Continuous innovations in this area are vital for fully harnessing the potential of CTCs in cancer diagnostics and therapeutic monitoring [98].

#### 3.1.2. Immunomagnetic Separation

Immunomagnetic separation can be positive or negative. Positive selection, as used in the FDA-approved CellSearch system, captures CTCs using magnetic beads coated with EpCAM-targeting antibodies. Immunomagnetic separation is a method that involves the use of magnetic beads coated with antibodies specific to antigens expressed on CTCs, such as epithelial cell adhesion molecules (EpCAM). When blood containing CTCs is passed over these beads, the CTCs adhere to the antibodies on the beads. A magnetic field is subsequently applied to isolate the CTC-bound beads from the rest of the blood sample [99].

Negative selection depletes blood cells, enriching CTCs indirectly by removing non-CTC components. This method is valuable for isolating both EpCAM-positive and EpCAM-low CTC subpopulations, aiding in cancer diagnosis and monitoring [46,77].

This method involves the use of antibodies to specifically target and capture CTCs that express the antigen of interest, leading to a highly specific and pure population of captured cells. The targeted antigen increases the likelihood of capturing CTCs of interest, ultimately contributing to the effectiveness of the method. Importantly, however, immunomagnetic separation may not capture CTCs that do not express the targeted antigen, potentially leading to false negative results. Furthermore, it is worth considering that this technique can incur significant costs owing to the expense of antibodies and magnetic beads, which should be factored into experimental planning and budgeting [46]. Recent scientific research has emphasised significant progress in immunomagnetic separation techniques. These advancements involve heightened sensitivity in detection methods and the creation of innovative antibodies specifically designed to target multiple antigens. This enables the capture of a broader spectrum of CTC subpopulations, leading to a more comprehensive understanding of the biological heterogeneity within these cells [100]. Integration with microfluidic devices has also facilitated more efficient processing of blood samples, reducing the time and costs associated with CTC isolation [101].

#### 3.1.3. Microfluidic Devices

These devices employ microscale channels and chambers to isolate CTCs from blood samples based on various physical properties, such as size, deformability, or immuno-affinity. Examples of such devices include the CTC chip and microfluidic vortex technology, which enhance the capture and isolation efficiency of CTCs [36].

These devices excel at capturing CTCs owing to their ability to process large blood sample volumes, isolating CTCs with high purity and yield. This purity and yield are crucial for downstream molecular analysis and clinical applications. However, microfluidic devices pose a primary challenge owing to the complexity involved in their fabrication and operation. Fabricating precise microscale channels and integrating functional components demands specialised expertise and equipment. In addition, microfluidic devices may face issues such as potential clogging of channels, which can impact their reliability and efficiency [36,102].

Recent research has concentrated on enhancing the performance and usability of microfluidic devices for isolating CTCs. Advancements in microfabrication techniques have allowed the creation of more durable and scalable microfluidic platforms capable of handling larger sample volumes and reducing processing times [103]. Integration with automated systems and sensitive detection methods has improved the practical utility of these devices in clinical settings, enabling real-time monitoring of CTC dynamics and treatment responses [36].

#### 3.1.4. Filtration-Based Methods

Filtration-based methods for isolating CTCs operate by exploiting the size disparity between CTCs and other blood cells. These methods typically utilise filters with specific pore sizes that allow smaller blood components to pass through while capturing larger CTCs. This size-based separation principle is effective in enriching CTCs from blood samples [104,105]. Filtration-based methods are valued for their simplicity and relatively low cost compared with other CTC isolation techniques. They do not require sophisticated instrumentation and can be implemented with basic laboratory equipment, making them accessible for research and clinical settings. Despite their simplicity, filtration-based methods may suffer from lower specificity than techniques that rely on molecular markers or physical properties such as deformability. There is a risk of potential damage to CTCs during the filtration process, which could affect their viability and downstream analysis [105].

Recent developments in filtration-based CTC isolation have focused on optimising filter materials and pore sizes to increase capture efficiency and purity. Advances in nanotechnology have allowed the production of filters with precisely controlled pore sizes, improving the specificity of CTC isolation while minimising damage to captured cells [77]. Integration with automated systems and real-time monitoring technologies has also facilitated the application of filtration-based methods in dynamic clinical environments, supporting rapid CTC detection and analysis.

#### 3.1.5. Dielectrophoresis (DEP)

Dielectrophoresis (DEP) is a technique that separates cells based on their dielectric properties when exposed to an electric field. CTCs, which exhibit electrical characteristics distinct from those of other blood cells, can be manipulated and isolated via this principle. DEP offers a label-free method of cell separation, preserving the integrity of live cells during the isolation process [106]. 

DEPs have a major benefit in that they do not require specific markers or labels on CTCs. This allows for the isolation and study of live CTCs, providing valuable insights into their biology and behaviour. DEP is also known for its ability to achieve high recovery rates of CTCs from blood samples, making it a promising tool for clinical applications. However, using DEP requires specialised equipment that can generate precise electric fields and control the sample conditions. Variations in sample conductivity and cell size can affect the efficiency and reliability of DEP, so it is important to carefully calibrate and optimise the process for consistent results in different experimental settings [7]. Advancements in dielectrophoresis (DEP) technology have focused on improving its efficiency and applicability in clinical settings. For example, the development of microfluidic DEP devices has made the isolation process more efficient, enabling rapid and high-throughput processing of blood samples [102]. Integration with automated systems and real-time monitoring capabilities has enhanced the reliability and reproducibility of DEP-based CTC isolation methods, making them suitable for routine clinical use.

#### 3.1.6. Polymerase Chain Reaction (PCR)-Based Methods

PCR-based methods play a pivotal role in the detection and characterisation of CTCs from blood samples, leveraging their unique genetic profiles. PCR amplifies specific genetic markers associated with CTCs, offering high sensitivity and specificity in identifying these rare cells amidst a background of normal blood cells [107]. This approach enables precise molecular profiling of CTCs, aiding in understanding their biological characteristics and potential clinical implications in cancer progression and treatment response [45,108].

Liquid biopsies encompass various tumour-derived materials shed into the bloodstream, including CTCs and circulating tumour nucleic acids such as ctDNA, ctRNA, exosomes, and other microvesicles [47,56]. ctDNA, which originates from apoptotic or necrotic tumour cells as well as CTCs, serves as a predictive biomarker for monitoring therapeutic response [109]. Notably, the FDA approval of the Cobas EGFR mutation test for managing lung cancer therapy is pivotal, underscoring the importance of ctDNA in cancer diagnosis and treatment decisions [110,111].

Various PCR methods have been developed to detect and characterise CTCs and ctDNA, continually advancing to improve sensitivity and enabling accurate quantitative analyses [112]. Compared with conventional methods, droplet digital PCR (ddPCR) represents a significant technological improvement, facilitating sample partitioning into numerous droplets to amplify rare mutations with enhanced sensitivity [113]. ddPCR also offers advantages such as reduced susceptibility to PCR inhibitors and the ability to perform absolute quantification without external calibration curves [114].

Real-time quantitative PCR (Q-PCR) allows for CTC quantification without nucleic acid extraction, simplifying workflows and enhancing efficiency [102]. Mei et al. demonstrated a robust detection method using tag-DNA-modified CK19 antibodies and EpCAM-conjugated magnetic beads, achieving a high detection rate in clinical samples and correlating CTC counts with tumour stage/status. The integration of single-cell and next-generation sequencing with PCR methods promises further advancements in CTC characterisation, elucidating their roles in cancer progression and treatment response [47,115].

The advantages of PCR-based methods include their ability to detect minimal quantities of CTCs and provide quantitative data on genetic alterations relevant to cancer prognosis and therapy [44]. These methods are particularly valuable for monitoring disease progression and treatment efficacy through the analysis of mutations, gene expression patterns, and chromosomal aberrations in CTCs [47,81,116]. However, PCR-based assays require prior knowledge of specific genetic markers associated with CTCs, limiting their utility in cases where these markers are unknown or heterogeneous [117].

Recent advancements in PCR technology, such as digital PCR (dPCR) and multiplex PCR assays, have increased the sensitivity and precision of CTC detection and characterisation [118]. These innovations allow for the detection of rare mutations and the quantification of CTCs with high accuracy, even in early-stage cancer patients [119]. Integrating next-generation sequencing (NGS) with PCR-based methods further expands their utility by providing comprehensive genomic and transcriptomic profiles of CTCs, revealing insights into tumour heterogeneity and evolution [108,120].

PCR-based methods represent a robust approach for the molecular analysis of CTCs, offering valuable insights into cancer biology, treatment response, and personalised medicine. Continued advancements in PCR technology and bioinformatics are expected to further enhance their clinical utility in oncology.

#### 3.1.7. Fluorescence-Activated Cell Sorting (FACS)

Fluorescence-activated cell sorting (FACS) is a sophisticated technique used to identify and isolate CTCs from blood samples. This method uses fluorescently labelled antibodies that bind specifically to CTC-specific antigens such as EpCAM, cytokeratins, or other surface markers found on tumour cells. The process involves passing the labelled blood sample through a flow cytometer, which detects and sorts cells on the basis of their fluorescence properties. FACS allows for the identification, quantification, and characterisation of CTCs, providing valuable insights into their molecular and phenotypic profiles [121].

The advantages of FACS include its high specificity and sensitivity in detecting rare CTCs among a large number of blood cells. Additionally, FACS can sort live cells for downstream analysis. This method enables the isolation of viable CTCs, which can be cultured or subjected to further molecular and functional assays to study their role in cancer progression and treatment response [122,123]. However, flow cytometry has several limitations, primarily related to its cost, complexity, and requirement for specialised instrumentation and skilled operators [91,103]. The initial setup and maintenance of a FACS system can be expensive, making it less accessible for routine clinical use in many healthcare settings. Moreover, the interpretation of FACS data requires expertise in flow cytometry, which may limit its widespread adoption without proper training and resources [91].

Recent advancements in FACS technology have focused on improving sensitivity, throughput, and automation to streamline the detection and analysis of circulating tumour cells (CTCs) [39,124]. New techniques involve the integration of microfluidic devices with FACS systems to increase efficiency and reduce sample processing times. Additionally, the development of novel fluorophores and multicolour panels has increased the range of markers that can be simultaneously analysed, allowing for more detailed CTC characterisation [37,124].

While FACS remains a powerful tool for CTC analysis owing to its specificity and ability to isolate live cells, ongoing technological advancements are necessary to address its cost and complexity barriers, thereby enhancing its clinical utility in cancer diagnosis, prognosis, and treatment monitoring.

#### 3.1.8. Immunofluorescence Microscopy

Immunofluorescence microscopy is a technique that uses fluorescently labelled antibodies to detect specific markers on CTCs. This allows for their identification and analysis through microscopic examination. This method enables researchers to visualise and confirm the morphology of CTCs, providing detailed insights into their structural characteristics [70]. One of the main advantages of IFM is its ability to offer high specificity through the use of antibodies that target distinct antigens expressed on CTCs, such as EpCAM or cytokeratins. This specificity allows for accurate differentiation of CTCs from other cells present in the blood. Additionally, the technique permits the simultaneous assessment of multiple markers via different fluorescent dyes, providing comprehensive phenotypic profiles of the isolated cells [122,123].

The method has several limitations. It is labour-intensive and requires meticulous sample preparation and careful handling to preserve cell integrity. The subjective nature of manual microscopy also poses challenges, as the identification and enumeration of CTCs can vary between observers, potentially leading to inconsistencies in data interpretation [70]. Moreover, IF microscopy is not well suited for high-throughput analysis, limiting its utility in studies that require processing large volumes of blood samples or analysing numerous patient samples on time [91,103].

Recent advancements in the field have focused on overcoming these limitations by integrating automated image analysis systems and machine learning algorithms. These innovations increase the accuracy and reproducibility of CTC detection and quantification [39,101,124]. For example, the development of automated fluorescence microscopes, along with sophisticated image processing software, enables rapid and unbiased identification of CTCs, thereby improving the method’s speed and reliability [81]. These advancements show promise in expanding the clinical applicability of immunofluorescence microscopy, making it a more robust tool for monitoring disease progression and therapeutic response in cancer patients [124].

While fluorescence microscopy remains a valuable technique for the detailed visualization and characterisation of CTCs, ongoing technological improvements are essential to overcome its inherent challenges and enhance its effectiveness for clinical and research applications.

#### 3.1.9. Nanoparticle-Based Methods

Nanoparticle-based methods for isolating and detecting CTCs have become valuable tools in cancer diagnostics and research. These methods involve the use of nanoparticles attached to specific antibodies or other ligands that bind to antigens on the surface of CTCs. Owing to the unique magnetic or optical properties of nanoparticles, it is possible to isolate and detect CTCs with high sensitivity and specificity. For example, magnetic nanoparticles can capture CTCs from blood samples by using an external magnetic field, allowing for efficient separation of CTCs from other blood components. Similarly, nanoparticles with optical properties, such as fluorescence, can help visualise and quantify CTCs via various imaging techniques [125].

Nanoparticle-based methods offer significant advantages in detecting CTCs because of their high sensitivity and specificity. By using antibodies or ligands that target specific CTC markers, these methods can capture only the relevant cells, minimise false positives, and improve detection accuracy. Furthermore, these techniques can be combined with other detection technologies, such as flow cytometry or molecular analysis, to provide comprehensive information about the captured CTCs. This integration allows for the genetic and protein-level characterisation of CTCs, providing valuable insights into tumour biology and potential therapeutic targets [95,126].

Despite their advantages, nanoparticle-based methods also face certain limitations. The synthesis and functionalization of nanoparticles can be complex and costly, requiring specialised equipment and expertise. The functionalization process, which involves attaching antibodies or ligands to the nanoparticles, must be carefully optimised to ensure effective binding to CTCs without compromising the stability or functionality of the nanoparticles. Additionally, the use of nanoparticles in clinical settings requires thorough evaluation of their biocompatibility and potential toxicity to ensure patient safety [127].

Recent advancements in nanoparticle technology have aimed to enhance the efficiency and precision of CTC capture and detection. For example, Lv et al. reported the use of clickable nanospheres for the effective capture and non-destructive release of CTCs. This innovative approach allows for the isolation of viable CTCs, which can then be further analysed or cultured, presenting numerous opportunities for downstream applications and research. Similarly, the development of multifunctional nanoparticles with combined magnetic and optical properties has facilitated the simultaneous capture and imaging of CTCs, thereby streamlining workflows and reducing analysis times [125,128].

Nanoparticle-based methods offer high sensitivity and specificity in isolating and detecting CTCs, making them promising approaches. Although challenges related to cost and complexity persist, continued research and technological advancements are expected to resolve these issues, leading to broader clinical adoption. The precision of CTC capture and analysis holds significant potential for improving cancer diagnosis, monitoring disease progression, and guiding personalised treatment strategies.

The methods for detecting CTCs are highly suitable for implementation as biomarker tools in identifying OSCC because of several key factors. These methods encompass various techniques enabling the isolation, detection, and analysis of CTCs from blood samples, providing valuable insights into cancer progression and therapeutic responses. The methodologies used include density gradient centrifugation, immunomagnetic separation, microfluidic devices, filtration-based methods, DEP, and PCR-based techniques, which offer specific and efficient ways to isolate and analyse CTCs. Additionally, FACS and immunofluorescence microscopy serve as effective methodologies for identifying and isolating CTCs in OSCC, offering high sensitivity and specificity. Recent technological advancements have further improved the efficiency and precision of capturing and detecting CTCs, thereby increasing their potential for integration into clinical practice.

### 3.2. Challenges and Limitations in CTC Detection and Enumeration

Detecting and counting CTCs in cancer patients, particularly those with OSCC, presents significant challenges and limitations. One of the primary obstacles is the exceedingly low abundance of CTCs in the bloodstream, often as few as one CTC per billion blood cells, necessitating highly sensitive and specific detection methods [47,129]. The heterogeneous nature of CTCs, reflecting the diversity of tumour cells within a primary tumour and its metastases, further complicates their detection. Variability in CTC surface markers, such as EpCAM, can result in false negatives if detection relies solely on one marker [116,130]. This is particularly relevant for OSCC, where EMT can cause CTCs to lose epithelial markers, making them less detectable by positive immunomagnetic separation or immunofluorescence techniques [131].

The purity of isolated CTCs presents a challenge. Techniques such as density gradient centrifugation and filtration-based methods may lead to contamination with other blood cells, reducing the accuracy of downstream analyses [127,129]. Moreover, some isolation methods, such as filtration, involve mechanical and shear forces that can damage CTCs, affect their viability, and compromise subsequent functional studies [105]. The complexity and cost of advanced detection platforms, such as microfluidic devices and Dielectrophoresis, restrict their widespread use in clinical settings, particularly in resource-limited environments [132].

In OSCC, detecting and characterising circulating tumour cells (CTCs) is more challenging because of the unique microenvironment of the tumour and the impact of local factors such as chronic inflammation and infection, which can change the characteristics of CTCs [128]. Additionally, the location of OSCC in the oral cavity results in the presence of various cell types and potential contaminants, requiring highly specific and reliable markers for the precise identification of CTCs [131].

Recent progress has been aimed at improving the detection and enumeration of CTCs. Technologies such as ddPCR and NGS offer increased sensitivity and specificity by targeting multiple genetic and epigenetic markers, thus accommodating the heterogeneity of CTCs [55,56,108]. Additionally, automated systems and integration with machine learning algorithms are being developed to increase the accuracy and reproducibility of CTC enumeration, reduce operator dependency, and increase throughput [132]. Despite these advancements, challenges remain in standardising CTC detection methods across different laboratories and ensuring their clinical utility in cancer management, including for OSCC. Continuous research and development are essential to overcome these limitations and to fully realise the potential of CTCs as biomarkers for early detection, prognosis, and monitoring of therapeutic responses in cancer patients.

While the sections above describe the available technologies for CTC detection, a critical comparison of these approaches is essential for determining their suitability for OSCC. Therefore, the following section evaluates the strengths, limitations, and OSCC-specific applicability of current CTC detection platforms, addressing methodological challenges and unresolved gaps in the field.

### 3.3. Critical Evaluation of CTC Detection Platforms: Strengths, Limitations, and Suitability for OSCC

While the section above summarises major technologies for CTC isolation and detection (Figure 3), a critical appraisal is necessary to determine their suitability for oral squamous cell carcinoma (OSCC), where phenotypic plasticity and epithelial–mesenchymal transition (EMT) is common. Below, we compare marker-based and marker-independent strategies, discuss inter-platform variability, and outline unresolved questions that currently limit routine clinical adoption in OSCC.

Marker-based (EpCAM-dependent) capture: standardisation vs. capture bias. EpCAM-dependent positive selection (exemplified by the FDA-cleared CellSearch^®^ workflow) remains the most standardised and widely validated approach to CTC enumeration in solid tumours, with robust analytical criteria, operator training frameworks, and a substantial body of clinical evidence in breast, prostate, and colorectal cancers [45,51,70]. These systems enable reproducible counts and facilitate between-centre comparisons, attributes that have historically accelerated translation in other diseases and that are attractive for multicentre OSCC studies [46]. However, reliance on epithelial markers introduces a capture bias: EpCAM/CK-low or negative CTCs frequent in EMT, hybrid EMT, and stem-like states may be under-detected, particularly relevant to OSCC where EMT programs are prominent [56,100]. Consequently, EpCAM-based enumeration can underestimate total CTC burden and miss clinically important mesenchymal/hybrid subpopulations that carry metastatic and therapy-resistant traits [57,91].

Marker-independent enrichment: broader phenotypic coverage with practical trade-offs. Marker-independent approaches size-based filtration, density gradients, deformability-based separation, dielectrophoresis (DEP), and microfluidics aim to capture a wider spectrum of CTC phenotypes, including EMT and cluster forms, and therefore often yield higher recovery of heterogeneous CTCs in head-and-neck contexts [95,104]. Size-based and density-based methods are relatively simple and low-cost but can suffer from contaminating leukocytes or CTC loss due to overlap in size/density with blood cells, which may reduce purity and complicate downstream molecular analyses [95,105]. DEP and microfluidic systems can increase yield and preserve viability for functional or single-cell multi-omic assays but may introduce device-specific learning curves, potential clogging, higher costs, and variability in recovery across platforms [36,99]. Notably, negative selection (CD45-based leukocyte depletion) enriches for both EpCAM-high and EpCAM-low CTC states and is therefore attractive for OSCC discovery studies, albeit with trade-offs in purity and standardisation [77].

OSCC-specific considerations: EMT, clusters, and clinical correlations OSCC cohorts demonstrate meaningful associations between CTC detection/levels and tumour stage, nodal involvement, or survival, but findings vary by platform and case mix [85,89]. Several studies highlight the presence of CTC clusters, minority events with disproportionate metastatic capacity whose detection is favoured by marker-independent capture and microfluidic workflows [19,65]. In contrast, small OSCC series using strictly epithelial markers report attenuated associations or post-operative signal fluctuations that may reflect capture bias rather than true biology [19,133]. Together, these observations argue for platform choice aligned to biological intent: (i) when the goal is harmonised enumeration for longitudinal surveillance or multicentre comparison, EpCAM-based systems offer standardisation; (ii) when the aim is to characterise EMT/hybrid states, clusters, or to enable downstream multi-omics, marker-independent or negative-selection microfluidics/DEP are preferable [90,100].

Inter-platform variability and analytical endpoints: A persistent barrier to clinical adoption in OSCC is the inter-platform variability in recovery, purity, and absolute counts—even from aliquots of the same specimen, driven by differing capture principles, gating criteria, and definition of a “CTC” [51]. Enumeration itself is sensitive to pre-analytical variables (tube type, time-to-processing, processed volume) and analytical thresholds (e.g., “≥1 CTC/7.5 mL,” “≥5 CTCs/7.5 mL”), which are not yet harmonised for OSCC. International expert groups have called for explicit reporting standards, fit-for-purpose validation, and context-of-use definitions to ensure that counts and molecular calls are comparable across laboratories and time [51,134]. Without this harmonisation, meta-analysis is difficult and prospective utility signals can be diluted.

Downstream characterisation: from enumeration to decision-enabling biology. One of the principal advantages of marker-independent capture is the compatibility with single-cell genomics/transcriptomics and high-content imaging, enabling characterisation of EMT, stemness, and drug-resistance programs that may not be apparent in archival tissue [92]. In OSCC and broader HNSCC, such molecular readouts are increasingly linked to risk of recurrence or progression and could inform trial designs that adapt therapy by CTC phenotype or genotype [89,135]. That said, workflow attrition (from capture to viable single cells to high-quality libraries) remains non-trivial and requires laboratory expertise and quality systems that are not yet ubiquitous in routine clinical settings [47].

Comparative suitability for OSCC use-cases. Screening/early detection (research): high-sensitivity marker-independent platforms can capture diverse phenotypes, but specificity and cost-effectiveness remain to be proven at population scale [95,104]. Staging/prognosis and surveillance: if comparability to historical literature is crucial, EpCAM-dependent enumeration provides standardisation; however, for OSCC where EMT is common, integrating a marker-independent stream (or negative selection) may reduce false-negative rates and improve risk attribution [85,100]. Therapy selection/monitoring: platforms preserving viability and enabling single-cell multi-omics (microfluidics/DEP/FACS) are favoured when the aim is to profile actionable pathways, immune markers, or evolving resistance [90,92,108].

Unresolved questions and gaps in OSCC-focused CTC research: First, head-to-head platform comparisons in OSCC are limited; prospective studies that randomise platform choice or process split samples in parallel are needed to quantify recovery bias and clinical signal across technologies [51]. Second, context-of-use-specific endpoints (e.g., CTC-defined minimal residual disease vs. phenotypic risk classifiers) and actionable thresholds have not been validated for OSCC, hindering clinician uptake [134]. Third, standardised pre-analytical/analytical SOPs—including blood volume, tube type, time-to-processing, and bioinformatics pipelines—are essential for reproducibility [123,129]. Fourth, integration with other liquid-biopsy analytes (ctDNA, EVs, miRNA) and imaging is likely to outperform any single biomarker; combined designs should be prioritised to improve sensitivity/specificity for recurrence and to deconvolute tumour evolution [28]. Finally, adequately powered multicentre OSCC cohorts with longitudinal sampling and predefined decision-impact analyses are required to move from association to clinical utility [89,134].

Pragmatic recommendation: For OSCC research and emerging clinical protocols, dual-track strategies are advisable: (i) an EpCAM-based enumeration track when harmonised counts are required (comparability, registries, mixed-tumour meta-analyses); and (ii) a marker-independent or negative-selection track when the priority is to capture EMT/hybrid states, clusters, and to enable downstream multi-omics that can inform precision therapy. Clear reporting of pre-analytical variables, capture conditions, and analytic thresholds, alongside adjudication by trained cytopathology or automated image analysis, will be essential to ensure reproducibility and to accelerate validation for OSCC [39,51,124]. This comparative analysis highlights key methodological limitations that continue to hinder accurate CTC detection in OSCC, providing a foundation for the challenges outlined in Section 4.

## 4. Clinical Relevance of CTCs in OSCC

Sample Biomarkers play crucial roles in the diagnosis, prognosis, and treatment of OSCC, offering valuable insights into disease progression, patient outcomes, and therapeutic responses. Recent advancements in biomarker research have contributed significantly to improving OSCC management strategies. For diagnosis, biomarkers serve as objective indicators of disease presence and severity, aiding clinicians in early detection and accurate classification of OSCC subtypes. Biomarker panels comprising molecular signatures, such as genetic mutations, protein expression profiles, and epigenetic alterations, have demonstrated utility in distinguishing OSCC from benign oral lesions and stratifying patients based on their risk of malignant transformation [136,137].

**Figure 4:**
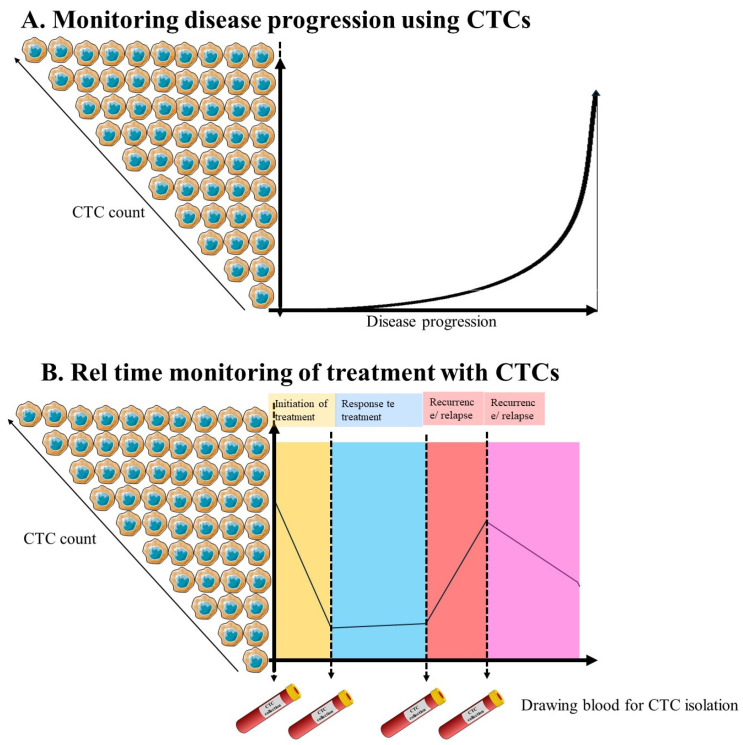
Clinical applications. Figure 4 illustrates the conceptual and investigational uses of role of circulating tumour cell (CTC) monitoring in cancer research. Panel (**A**) shows how CTC counts increase with disease progression, highlighting its potential as a biomarker for early detection and metastasis assessment. Panel (**B**) demonstrates real-time treatment monitoring, where a decline in CTC levels could indicate a positive response, while subsequent increases may signal recurrence or relapse. By periodically analysing CTCs through liquid biopsy, it may be possible for clinicians to assess treatment efficacy, detect early signs of disease progression, and make timely therapeutic adjustments outcomes.

Furthermore, biomarkers hold promises for predicting OSCC prognosis by identifying patients at increased risk of disease recurrence, metastasis, or treatment resistance. Prognostic biomarkers, including tumour size, nodal involvement, and histopathological features, inform decision-making treatment and enable personalised care strategies tailored to individual patient needs [138,139] (Figure 4A,B).

In addition to their diagnostic and prognostic roles, biomarkers play a pivotal role in guiding OSCC treatment selection and monitoring therapeutic responses. Biomarker-driven approaches facilitate the identification of targetable molecular pathways, and the development of precision medicine interventions aimed at maximising treatment efficacy while minimising adverse effects [139,140].

### 4.1. Potential Applications of CTCs as Diagnostic, Prognostic, and Predictive Biomarkers

Circulating tumour cells are highly important in cancer research and clinical applications because of their potential to provide valuable insights into various aspects of cancer biology and patient care. Recent advancements in CTC research have notably advanced our understanding of their roles in cancer progression, metastasis, treatment response, and personalised medicine. These recent developments carry substantial clinical implications that have the potential to revolutionise various aspects of cancer [21].

The clinical significance of CTCs in OSCC is increasingly acknowledged, with potential applications as diagnostic, prognostic, and predictive biomarkers. These cells provide a non-invasive avenue for gaining insights into tumour dynamics and disease progression (Figure 4A), which could play a pivotal role in enhancing patient management and improving outcomes.

Diagnostic biomarkers: Compared with conventional imaging and biopsy methods, CTCs have the potential to serve as early diagnostic markers for OSCC, facilitating the detection of malignancy at an earlier stage. This is particularly crucial in OSCC, where early detection significantly improves prognosis. Studies have demonstrated that CTCs can be identified in the blood of patients with OSCC and are correlated with the presence of primary tumours [39]. Advances in technologies such as liquid biopsy allow for the detection of CTCs and their genetic alterations, offering a more comprehensive overview of the disease landscape [47].

Prognostic Biomarkers: The presence and quantity of CTCs in the bloodstream are correlated with prognosis in OSCC patients. Higher counts of CTCs are often associated with advanced disease stages, lymph node involvement, and a greater likelihood of metastasis, all of which contribute to poorer outcomes [85]. This trend is consistently reflected across OSCC cohorts, as shown in Table 1, where multiple independent studies demonstrate that CTC presence, quantity, or phenotype correlates strongly with tumour stage, nodal involvement, survival, and metastatic behaviour. Moreover, specific characteristics of CTCs, such as the expression of EMT markers, can provide additional prognostic information, indicating more aggressive tumour behaviour and potential resistance to therapy [127].

Predictive biomarkers: CTCs can also be valuable in predicting therapeutic response and resistance. By analysing the genetic and molecular profiles of CTCs, clinicians can identify mutations and alterations that drive resistance to specific treatments, thereby enabling more personalised therapeutic approaches. For example, the detection of epidermal growth factor receptor (EGFR) mutations in CTCs can guide the use of targeted therapies in OSCC patients [141,142]. Additionally, monitoring changes in CTC counts and characteristics during treatment can provide real-time insights into treatment efficacy, allowing timely adjustments to therapeutic strategies [141]. 

**Table 1:** This shows the value of CTCs in OSCC management.

Study	Sample Size	Isolation Technique	Tumour Site	Findings
[84]	80	CellSearch system	OSCC	Detection of CTC was correlated with advanced tumour and distant metastasis.
[143]	40	Laser scanning cytometry	OSCC	High levels of CTC were associated with poor survival.
[86]	93		OSCC	CTC—positive correlation with tumour stage, nodal stage, metastatic and clinical stage.
[88]	50	Nanovelcro System	Tongue	CTCs—positive correlation with clinical stage and nodal. CTC were independent predictors of disease-free survival and overall survival.
[85]	152	OncoDiscover of CTCs	OSCC	CTC prognostic survival and disease progress. CTC correlated with tumour stage and nodal stage.
[87]	22	Immunohistochemistry	OSCC	No correlation between CTC, CTM and metastatic event. Increase of CTC and CTM after surgery although it was not statistically significant.
[19]	13	Microfluidic chip	OSCC	No correlation between CTC with metastatic events. CTC clusters were associated with metastatic OSCC.
[144]	85	Negative enrichment and immunostaining and Tongue Fluorescence in situ hybridisation		No difference in preop and post CTC during disease progression. Postoperative CTC is independent risk factor for tongue squamous cell carcinoma.

Recent Advances and Applications: Recent studies have explored the use of various CTC detection methods, such as microfluidic devices, dielectrophoresis, and next-generation sequencing, to increase sensitivity and specificity [101]. These advancements are making it increasingly feasible to integrate CTC analysis into routine clinical practice for OSCC. For example, a recent study demonstrated the utility of a microfluidic liquid biopsy platform (CT elect) for automated isolation and enrichment of CTCs from HNSCC (including OSCC) patient blood, supporting its potential for clinical monitoring and personalised management [90]. The integration of CTC analysis into the clinical management of OSCC holds promise for enhancing diagnostic accuracy, predicting disease progression, and tailoring individualised treatment plans. Continuous research and technological advancements are essential to overcome current challenges and fully harness the potential of CTCs as clinical biomarkers in OSCC.

CTCs have emerged as crucial biomarkers in cancer research and clinical practice, offering valuable insights into various facets of cancer biology and patient care. Recent advancements underscore their importance in early cancer detection, prognostication, treatment response monitoring, and personalised medicine. Early cancer detection benefits significantly from CTCs, enabling liquid biopsy-based screening that identifies cancers at earlier stages, increasing treatment efficacy and patient outcomes [70]. CTCs not only predict disease occurrence but also aid in distinguishing healthy individuals from those with cancer or precancerous conditions with high sensitivity before conventional diagnostic methods [100,145]. Prognostically, higher CTC counts and the presence of CTC clusters correlate with poorer outcomes across various cancers, guiding treatment decisions and predicting patient survival [146,147]. The heterogeneity of CTCs reflects the diversity within primary tumours, providing insights into disease progression and therapeutic response variability. CTCs are integral in monitoring metastatic potential, facilitating early intervention strategies and personalised treatment plans based on metastatic risk assessments [133]. Ignatiadis & Dawson discuss how CTC and ctDNA analysis captures tumour evolution during treatment, supporting their use as dynamic biomarkers for treatment response and clinical decision-making [148]. The detection of peripheral blood CTCs is associated with worsened outcomes and may inform MRD and therapeutic interventions after treatment [148]. Molecular profiling of CTCs can uncover genetic differences not seen in primary tumours, elucidating mechanisms of therapy resistance and informing personalised treatment strategies [149]. Also, genomic profiling studies have shown reproducible serial genomic changes in CTCs over time during therapy, reinforcing their utility for molecular monitoring of response [150]. Bidard and colleagues summarise clinical evidence for using CTC counts and molecular features to guide treatment choices and support trial designs testing CTC-guided therapeutic strategies [151].

### 4.2. Correlations Between CTC Presence/Quantity and OSCC Stage, Grade, and Metastatic Potential

Understanding the correlation between CTCs and oral squamous cell carcinoma (OSCC) stage, grade, and metastatic potential provides critical insights into disease progression and prognosis. Recent studies have shown that the presence and quantity of CTCs are significantly correlated with the clinical characteristics of patients with OSCC, reflecting disease severity and metastatic propensity. OSCC, characterised by aggressive local invasion and potential metastasis, involves varying degrees of CTC shedding into the bloodstream, which can serve as indicators of disease stage and progression [85]. High CTC counts have been associated with advanced stages of OSCC, including larger primary tumours and lymph node involvement, highlighting their utility as prognostic markers [86]. Additionally, CTC enumeration has demonstrated predictive value in identifying OSCC patients at increased risk of metastasis, guiding treatment decisions and posttreatment monitoring strategies [85].

Recent advancements in CTC detection technologies, such as microfluidic devices and immunomagnetic isolation methods, have increased the sensitivity and specificity of CTC detection in OSCC patients [77]. These techniques allow for the characterisation of CTCs based on molecular markers associated with tumour aggressiveness and metastatic potential, providing deeper insights into disease biology. For example, studies have identified specific CTC phenotypes in OSCC that correlate with tumour grade and recurrence rates, underscoring their role in monitoring disease progression and treatment response [135].

Furthermore, the presence of CTC clusters, a more aggressive form of CTCs associated with increased metastatic potential, has been observed in OSCC patients and linked to poorer clinical outcomes [86]. These clusters not only facilitate the dissemination of tumour cells but also contribute to the establishment of distant metastases, highlighting their significance as prognostic indicators in OSCC [135].

Furthermore, CTCs play a pivotal role in assessing OSCC stage, grade, and metastatic potential, offering valuable insights into disease progression and prognosis. Advances in CTC detection and characterisation continue to refine their clinical utility, paving the way for personalised treatment strategies and improved patient outcomes in patients with OSCC.

### 4.3. Impact of CTC Detection on Treatment Selection and Monitoring

The impact of detecting CTCs on treatment selection and monitoring in cancers, including OSCC, is increasingly recognised for its potential to guide personalised therapeutic approaches and improve patient outcomes. Recent studies have shown that CTC detection provides crucial insights into treatment response and disease progression in patients with OSCC, influencing clinical decision-making and therapeutic strategies. CTCs serve as dynamic biomarkers that reflect the heterogeneous nature of OSCC tumours, aiding in the selection of targeted therapies and monitoring treatment efficacy [135].

In OSCC, the presence and enumeration of CTCs have been correlated with treatment response and disease recurrence, offering valuable prognostic information beyond conventional clinical parameters. High CTC counts before and during treatment have been associated with poorer outcomes, including increased risk of metastasis and reduced overall survival rates, emphasising their role in predicting treatment resistance and disease aggressiveness. This predictive value enables oncologists to tailor treatment regimens more precisely, optimising therapeutic efficacy and minimising unnecessary interventions [85,135].

Advancements in CTC detection technologies, such as microfluidic devices and immunomagnetic isolation methods, have enhanced the sensitivity and specificity of CTC analysis in OSCC [85]. These techniques allow real-time monitoring of CTC dynamics throughout the course of treatment, facilitating early detection of treatment resistance or disease recurrence [86]. For example, studies have demonstrated that changes in CTC counts and molecular profiles correlate with treatment response in OSCC patients undergoing chemotherapy or targeted therapies, enabling prompt adjustments to treatment plans [151].

Furthermore, CTC-based liquid biopsies provide a non-invasive alternative to traditional tissue biopsies, offering a real-time assessment of tumour progression and molecular evolution [85]. The molecular characterisation of CTCs, including the analysis of genetic mutations and expression profiles, can inform clinicians about potential therapeutic targets and mechanisms of resistance in OSCC [20,89]. This personalised approach to cancer management based on CTC analysis holds promise for improving treatment outcomes and survival rates in OSCC patients.

Ultimately, CTC detection plays a pivotal role in guiding treatment selection and monitoring in OSCC, facilitating personalised therapeutic strategies and enhancing clinical decision-making. Ongoing research and technological innovations continue to expand the utility of CTC-based assays in improving patient outcomes and advancing precision oncology.

### 4.4. Molecular Profiling of CTCs for Precision Oncology in OSCC

Molecular profiling of circulating tumour cells (CTCs) has become an essential component of precision oncology, providing a dynamic method to characterise tumour evolution non-invasively. In oral squamous cell carcinoma (OSCC), where tumours are highly heterogeneous, CTCs offer a valuable real-time representation of the molecular alterations underpinning disease progression. Comparative studies demonstrate that CTCs often exhibit molecular features distinct from those observed in primary tumour tissue. For example, transcriptomic analyses in OSCC show that CTCs cluster separately from their matched primary tumours, revealing marked genomic and phenotypic divergence that underscores the limitations of single-site tissue biopsies [135]. Broader head and neck squamous cell carcinoma (HNSCC) research further support this discordance, showing that circulating analytes, including CTCs and circulating tumour DNA (ctDNA), capture mutations and copy-number variations absent in the original tumour sampling, reflecting tumour evolution and clonal selection over time [152].

Beyond genomic discordance, CTCs can reveal actionable therapeutic mutations that guide individualised treatment. Although OSCC-specific CTC mutation research remains limited, evidence from HNSCC liquid biopsy studies highlights recurrent alterations in genes such as *TP53*, *PIK3CA*, *CDKN2A*, *EGFR*, and *NOTCH1*, all of which are relevant to OSCC biology and therapeutic decision-making [152]. These mutations influence essential pathways related to proliferation, apoptosis, and response to targeted agents, and their emergence in CTCs may help clinicians select targeted therapies, including EGFR inhibitors or PI3K/AKT pathway modulators, earlier in the treatment process. Complementing this, OSCC-specific CTC transcriptomic profiling demonstrates significant heterogeneity across epithelial, mesenchymal, and stemness-related signatures (e.g., *EpCAM*, *EGFR*, *VIM*), suggesting that CTC molecular features may serve as biomarkers for predicting disease aggressiveness and treatment response [153].

Immune-related biomarkers represent another important domain where CTC molecular profiling can guide precision therapy. Within HNSCC, PD-L1 expression on CTCs has emerged as a promising predictor of response to immune checkpoint inhibitors, given that PD-L1 expression may fluctuate across time and tumour sites [152,153]. Although OSCC-specific data are sparse, the immunological mechanisms described in OSCC, such as epithelial–mesenchymal transition (EMT), platelet cloaking, and inflammatory microenvironment modulation closely mirror those in HNSCC, suggesting comparable relevance of PD-L1-positive CTCs in immunotherapy planning. Because PD-L1 expression can evolve during treatment, CTC-based immune profiling provides a non-invasive means of tracking dynamic immunological states, potentially supplementing or even replacing static tissue-based immunohistochemistry in the future.

CTC profiling is also increasingly recognised for its capacity to monitor emerging resistance mechanisms. Serial sampling of OSCC CTCs reveals extensive transcriptomic heterogeneity, identifying distinct CTC subclusters associated with poorer overall and disease-free survival, an indication that molecular adaptations within CTCs reflect evolving therapeutic resistance [153]. Furthermore, OSCC studies incorporating both CTC and cfDNA analysis report that elevations in CTC cluster counts and increased preoperative cfDNA levels may signal an elevated risk of metastasis or recurrence [19]. This highlights the potential of integrating CTC-based molecular profiling with clinical and imaging assessments to detect treatment failure earlier and to personalise therapy adjustments.

Overall, molecular characterisation of CTCs, including genomic, transcriptomic, and immune-related profiling, represents a transformative tool for precision oncology in OSCC. By capturing tumour heterogeneity more comprehensively than static tissue biopsies, CTC-based assays facilitate early detection of actionable mutations, immune biomarkers, and resistance pathways. As technological advancements continue to expand the feasibility and fidelity of CTC analyses, integrating these liquid biopsy approaches into OSCC management will likely enhance early detection, improve treatment stratification, and support adaptive therapeutic strategies.

## 5. Future Perspectives and Challenges: Need for Standardised Multicentre Studies with Large Sample Sizes to Assist in Validation of Biomarkers in OSCC

Despite the promise of CTC-based liquid biopsies, several challenges remain. Owing to the rarity and heterogeneity of CTCs in blood samples, their detection and analysis are technically demanding. Researchers are continually working to refine enrichment and isolation techniques to increase the sensitivity and specificity of CTC detection. Additionally, integrating CTC analysis with other clinical data, such as tumour marker, imaging, and patient history data, can increase the overall diagnostic and prognostic value of this approach [20]. The detection and analysis of CTCs through liquid biopsies represents a significant advancement in the field of oncology. This minimally invasive method provides a valuable tool for monitoring cancer progression, assessing treatment response, and detecting early signs of metastasis. Ongoing research and technological developments are expected to further improve the clinical utility of CTCs, making them an integral part of personalised cancer care [154].

CTCs are present in extremely low numbers compared with other blood cells, making their isolation a challenging task akin to finding a needle in a haystack. Advanced technologies with high sensitivity are therefore essential for efficiently capturing and detecting these rare cells. Real-time monitoring of CTC dynamics, capturing changes in CTC numbers and characteristics over time, is crucial for understanding the temporal aspects of cancer progression, treatment response, and the emergence of resistance [41,127].

The development of new high-throughput technologies allows for the rapid and automated processing of large volumes of blood, significantly enhancing the efficiency of CTC isolation. This is particularly important for clinical applications where timely results are essential for making informed treatment decisions [155]. However, integrating CTC research findings into routine clinical practice requires a concerted effort toward standardization, validation, and collaboration between researchers and healthcare professionals. Validation of CTC-based assays is essential to ensure their reliability and reproducibility across different settings and patient populations [149].

To make CTC analysis clinically relevant, existing or new technologies need to be adaptable to routine clinical practice. This involves developing user-friendly platforms that can be seamlessly integrated with existing diagnostic workflows and point-of-care systems [102,156,157]. The integration of advanced analytical platforms, such as single-cell sequencing and next-generation sequencing, will enable comprehensive profiling of CTCs, providing detailed insights into their genetic and molecular characteristics. These technologies also increase accessibility while significantly reducing the time and costs associated with CTC analysis [158]. The use of CTCs in cancer diagnosis, prognosis, and monitoring treatment outcomes has the potential to revolutionise our approach to cancer detection and treatment. By enabling continuous monitoring of tumour dynamics and treatment responses, CTC-based approaches can provide real-time insights that help tailor therapies to individual patients’ needs. This personalised approach can lead to more effective treatments, improved patient outcomes, and potentially lower healthcare costs by avoiding ineffective therapies and reducing the need for more invasive diagnostic procedures [47].

The efficient isolation and detailed analysis of CTCs are crucial for advancing cancer care. The ongoing development of high-sensitivity and high-throughput technologies, combined with efforts to integrate these advancements into clinical practice, will pave the way for CTCs to become a cornerstone of personalised oncology. By harnessing the full potential of CTCs, we can improve early detection, refine prognostic assessments, and optimise treatment strategies, ultimately enhancing the quality of care for cancer patients.

Understanding the specific molecular changes in CTCs associated with OSCC holds significant promise for advancing personalised therapeutic strategies. Recent research has revealed various molecular alterations in CTCs that are correlated with OSCC progression and treatment response, offering insights into potential therapeutic targets. For example, studies have shown that CTCs from OSCC patients frequently exhibit genetic mutations in key oncogenes and tumour suppressor genes, such as TP53, EGFR, and PTEN, which play critical roles in cancer pathogenesis and treatment resistance [28,159,160]. These molecular profiles reflect the heterogeneity of OSCC tumours and provide valuable information for tailoring targeted therapies based on individual genetic profiles. Additionally, analysis of gene expression patterns in CTCs has revealed dysregulation of signalling pathways involved in cell proliferation, apoptosis evasion, and EMT, which are crucial for OSCC metastasis and resistance to therapy [159].

This molecular characterisation of CTCs not only enhances our understanding of OSCC biology but also enables clinicians to monitor disease progression and predict treatment response in real time (Figure 4B). The incorporation of CTC-based liquid biopsies into clinical practice allows early detection of treatment resistance and disease recurrence, guiding timely adjustments to therapeutic regimens to improve patient outcomes in patients with OSCC [19,160,161]. Therefore, leveraging the molecular insights gained from CTC analysis holds immense potential for developing personalised treatment strategies that target specific molecular aberrations that drive OSCC progression and resistance.

The integration of CTC-based diagnostics and therapies into standard clinical practice for managing OSCC is progressively advancing with emerging technologies and recent advancements in CTC research. Technological innovations, such as microfluidic devices and high-resolution imaging techniques, have increased the sensitivity and specificity of CTC detection in OSCC patients, enabling early detection and monitoring of disease progression [134,162]. These advancements are pivotal in personalised medicine, where molecular characterisation of CTCs informs targeted therapies tailored to individual genetic profiles, thereby improving treatment efficacy and patient outcomes [77]. However, challenges persist, including CTC heterogeneity and rarity, necessitating robust methodologies for isolation and characterisation to capture the full spectrum of OSCC disease biology [162]. Standardising detection methods across different platforms and clinical settings is crucial to ensure the reproducibility and reliability of CTC-based assays in routine clinical practice. This will help to overcome current limitations and facilitate widespread adoption [159,160,162]. As these technologies continue to evolve and clinical validation studies progress, integrating CTC-based diagnostics and therapies holds promise for revolutionising the management of OSCC and moving towards more precise and effective patient care strategies.

## 6. Conclusions

Circulating tumour cells (CTCs) represent a biologically and clinically significant component of liquid biopsy, offering a minimally invasive means to assess tumour behaviour, metastatic potential, and treatment response. In OSCC, CTCs provide valuable insights into tumour heterogeneity, epithelial–mesenchymal plasticity, stemness, and evolving resistance mechanisms. However, their application remains constrained by technical challenges, including extreme rarity, phenotypic diversity, and platform-dependent variability in detection and enumeration.

Although CTC enumeration is clinically validated in several solid tumours, its utility in OSCC is yet to be fully established. Technological advances, including microfluidic enrichment, dielectrophoresis, negative-selection workflows, and high-resolution molecular profiling, have improved the sensitivity and breadth of detectable CTC phenotypes. Nevertheless, enumeration alone remains insufficient due to low abundance and the underrepresentation of EMT-shifted or EpCAM-low CTCs. Multimodal strategies that incorporate molecular characterisation, including genomic, transcriptomic, and immunophenotypic profiling, offer a more comprehensive assessment and may better capture clinically actionable information.

Integrating CTC analysis with complementary biomarkers such as ctDNA, exosomes, and miRNAs, as well as conventional clinical parameters and imaging, provides a more robust framework for early detection, prognostic assessment, and therapy monitoring in OSCC. To advance towards routine clinical implementation, standardised pre-analytical and analytical procedures, inter-platform harmonisation, and large, well-designed multicentre validation studies are essential.

In summary, while significant progress has been made, further work is required to fully realise the clinical promise of CTCs in OSCC. Continued technological refinement, standardisation, and integration with multi-analyte liquid biopsy approaches will be critical for establishing CTCs as reliable biomarkers capable of enhancing early diagnosis, personalising treatment, and improving patient outcomes.

## Data Availability

No new data were created or analyzed in this study. Data sharing is not applicable to this article.
